# Prospective Evaluation of *MGMT*-Promoter Methylation Status and Correlations with Outcomes to Temozolomide-Based Chemotherapy in Well-Differentiated Neuroendocrine Tumors

**DOI:** 10.3390/curroncol30020106

**Published:** 2023-01-18

**Authors:** Nicole Brighi, Giuseppe Lamberti, Elisa Andrini, Cristina Mosconi, Lisa Manuzzi, Giada Donati, Andrea Lisotti, Davide Campana

**Affiliations:** 1 Department of Medical Oncology, IRCCS Istituto Romagnolo per lo Studio dei Tumori (IRST) “Dino Amadori”, 47014 Meldola, Italy; 2 Department of Experimental, Diagnostic and Specialty Medicine, Sant’Orsola-Malpighi University Hospital, ENETS Center of Excellence, 40138 Bologna, Italy; 3 Division of Medical Oncology, IRCCS Azienda Ospedaliero-Universitaria di Bologna, Via P. Albertoni 15, 40138 Bologna, Italy; 4 Department of Radiology, IRCCS Azienda Ospedaliero-Universitaria di Bologna, 40138 Bologna, Italy; 5 Gastroenterology Unit, Hospital of Imola, University of Bologna, 40026 Bologna, Italy

**Keywords:** neuroendocrine neoplasms, chemotherapy, biomarkers, epigenetic, gastroenteropancreatic

## Abstract

Temozolomide (TEM) as a single agent or in combination with capecitabine (CAPTEM) is active in well-differentiated advanced neuroendocrine tumors (NETs) of gastro-entero-pancreatic and thoracic origin. The predictive role of MGMT-promoter methylation in this setting is controversial. We sought to prospectively evaluate the MGMT-promoter methylation status ability to predict outcomes to TEM-based chemotherapy in patients with NET. A single-center, prospective, observational study has been conducted at the ENETS Center-of-Excellence Outpatient Clinic of the IRCCS Policlinico Sant’Orsola-Malpighi in Bologna, Italy. Patients with advanced, gastro-entero-pancreatic or lung well-differentiated NETs candidate to TEM-based chemotherapy and with available tumor samples for MGMT-promoter methylation assessment were included. The MGMT-promoter methylation status was analyzed by using pyrosequencing. The primary endpoint was progression-free survival (PFS) by the MGMT-promoter methylation status. Secondary endpoints included overall survival (OS), objective response rate (ORR), disease control rate (DCR), and safety. Survival outcomes were compared by restricted mean survival time (RMST) difference. Of 26 screened patients, 22 were finally enrolled in the study. The most frequent NET primary sites were the pancreas (64%) and the lung (23%). MGMT promoter was methylated in five tumors (23%). At a median follow-up time of 47.2 months (95%CI 29.3–89.7), the median PFS was 32.8 months (95%CI 17.2–NA), while the median OS was not reached. Patients in the methylated MGMT group, when compared to those in the unmethylated MGMT group, had longer PFS (median not reached [95%CI NA–NA] vs. 30.2 months [95%CI 15.2–NA], respectively; RMST *p* = 0.005) and OS (median not reached [95%CI NA–NA] vs. not reached [40.1–NA], respectively; RMST *p* = 0.019). After adjusting for confounding factors, the MGMT-promoter methylation status was independently associated to the PFS. Numerically higher ORR (60% vs. 24%; *p* = 0.274) and DCR (100% vs. 88%; *p* = 1.00) were observed in the methylated vs. unmethylated MGMT group. TEM-based chemotherapy was well-tolerated (adverse events grade ≥3 < 10%). In this prospective study, MGMT-promoter methylation predicted better outcomes to TEM-based chemotherapy in patients with NET.

## 1. Introduction

Temozolomide is an orally-available alkylating agent active in advanced neuroendocrine tumors (NETs), both as monotherapy (TEM) or in association with capecitabine (CAPTEM). Studies have reported objective response rates (ORR) with TEM ranging from 30 to 70%, with even higher rates in combination-therapy studies, in particular with CAPTEM, in pancreatic NETs (panNETs) [1,2,3]. On the other hand, TEM-treatment efficacy seems to be lower in intestinal NETs, with an ORR of 7% [4]. 

Current guidelines recommend the use of TEM in monotherapy or in association for the treatment of advanced midgut, thoracic, and panNETs [5,6,7,8]. However, there is no guide for clinicians to choose TEM over other treatments or to sequence and improve patient selection. 

The DNA damage dealt by TEM is repaired by the “suicide” enzyme O6-methylguanine-DNA methyltransferase (MGMT) [9]. Because of its mechanism of action, the intracellular quantity of MGMT, which is regulated by gene-promoter methylation, is inversely correlated with TEM activity [10]. Among the different assays available, pyrosequencing is the most used to assess *MGMT*-gene-promoter methylation [11,12]. The correlation of the *MGMT*-promoter methylation status with response to TEM has been proved in brain tumors, and its assessment is standard in gliomas [13,14,15,16]. Nevertheless, data about the role of this biomarker in NETs are still debated because conflicting results have been reported. However, a prospective trial showed that the combination of capecitabine plus temozolomide was associated with a significant improvement in PFS compared to temozolomide alone in advanced pancreatic NEN patients [17]. This can be due to the retrospective nature of these studies and the different methods used to assess *MGMT* silencing, including promoter sequencing and immunohistochemistry (IHC) [18,19,20,21,22,23,24,25,26]. 

Because of the impact in the treatment of NETs, we sought to investigate prospectively the role of the *MGMT*-promoter methylation status as a predictive biomarker of response to TEM-based chemotherapy in patients with advanced well-differentiated gastro-entero-pancreatic (GEP) and thoracic NETs.

## 2. Materials and Methods

### 2.1. Study Design 

A single-center, prospective, observational study was conducted at the ENETS Center-of-Excellence Outpatient Clinic of the IRCCS Policlinico Sant’Orsola-Malpighi of Bologna (Italy). Between November 2018 and October 2021, well-differentiated gastro-entero-pancreatic or thoracic NETs (WD-NET) of all patients who were candidate to receive TEM-based chemotherapy were tested for MGMT-promoter methylation status before treatment start and were followed-up according to clinical practice. Data-lock date was October 15, 2022. All patients provided written informed consent for treatment and for all the procedures related to the study. This study was approved by a local IRB (Comitato Etico Indipendente, IRCCS Policlinico Sant’Orsola-Malpighi of Bologna) and was conducted in accordance with the principles of the Declaration of Helsinki (revision of Edinburgh, 2000).

### 2.2. Study Population

All consecutive patients responding to the inclusion criteria were included. Inclusion criteria were: age ≥18 years; performance status by Eastern Cooperative Oncology Group (ECOG PS) 0–1; well-differentiated NETs (GEP) and typical or atypical carcinoids (thoracic), according to WHO’s 2019 classification [27]; grading 1-2-3, according to WHO’s 2019 classification; primary site (pancreas, gastro-intestinal tract, lung); locally advanced (III) or metastatic (IV) stage; and availability of tissue for *MGMT*-promoter methylation status analysis (formalin-fixed, paraffin-embedded tissue) from biopsy or surgical resection of tumor (primary or metastasis). Patients had to be considered by clinician’s choice as candidates for TEM-based treatments with either TEM alone (180–200 mg/mq day 1–5 every 4 weeks) or CAPTEM (capecitabine 1500 mg/sqm day 1–14 in two daily doses and TEM 180–200 mg/sqm day 10–14, every 4 weeks) as indicated by clinical guidelines [5,6,7,8].

### 2.3. Data Collection

Demographic, clinical, molecular, and pathological data were prospectively collected. A computerized data sheet was created and updated at each visit. For each patient, the following data were collected: age, sex, date of diagnosis, age at diagnosis, presence of MEN1 syndrome, presence of functioning syndrome, pathological features (tumor primary site, grading, Ki-67 value, WHO’s 2019 classification, and TNM staging according to ENETS), previous treatments (type and time to progression), TEM-based treatment data (regimen, doses, treatment line, start and discontinuation date, reason for discontinuation, cycle number, and concomitant medications), adverse events (grading per Common Terminology Criteria for Adverse Events [CTCAE] v.5.0, correlation with treatment, date of onset, and resolution), outcome data (date of progression, death date, best response, and date of best response), and molecular data (presence of MGMT methylation). Treatment regimen (TEM in monotherapy or in association with capecitabine) was established by the treating physician’s choice. Computed tomography (CT) scans were performed at baseline and every 3 months (±1 month) until disease progression according to RECIST v1.1 criteria (unless clinical conditions required shorter intervals) [28]. CT scans were performed by a NEN-expert radiologist of the Bologna ENETS Center of Excellence (C.M.).

### 2.4. MGMT-Promoter Methylation Status Analysis

The analysis was performed at the Molecular Pathology Laboratory at IRCCS Policlinico Sant’Orsola-Malpighi of Bologna. *MGMT*-promoter methylation status was evaluated using pyrosequencing. To be considered fully evaluable, the samples had to contain more than 80% tumor cells. DNA extraction from formalin-fixed, paraffin-embedded tissue (from surgical resection specimen or biopsy of primary tumor or metastases) was performed after deparaffinization using a purification kit (MasterPure DNA, Epicentre, Madison, WI, USA). Genomic DNA was modified by bisulfite conversion (EZ DNA Methylation Gold Kit, Zymo, Irvine, CA, USA). Pyrosequencing was performed using the PyroMark Q24 CpG MGMT kit (Qiagen, Hilden, Germany) on a PyroMark Q24 System (Qiagen). Data were analyzed and quantified with the PyroMark Q24 Software 2.0.7 (Qiagen). The mean percentage of the five CpG methylated islands detected by the kit was used for analysis. An 8% cut-off was used, accordingly to neuro-oncology clinical practice: *MGMT* was considered methylated if methylated alleles were more numerous than not-methylated alleles by at least 8%; otherwise *MGMT* was scored as not methylated [29,30].

### 2.5. Study Objectives and Endpoints 

The primary objective of the study was to evaluate the role of MGMT-promoter methylation status in predicting the response to TEM-based regimens in NETs. The primary endpoint of the study was progression-free survival (PFS) by MGMT-promoter methylation status. Secondary objectives of the study include the evaluation of activity by MGMT-promoter methylation status and safety of TEM-based regimens. Secondary endpoints were thus objective response rate (ORR), disease control rate (DCR), and overall survival (OS). Safety of TEM-based treatments was assessed by monitoring any adverse events (AEs). Finally, an analysis to evaluate the costs of this analysis and its feasibility in clinical practice was performed. PFS was measured as the time from treatment start to radiological progression according to RECIST v1.1 or death by any cause, whichever occurred first, while OS was measured as the time from treatment start to death by any cause. ORR and DCR were the rate of the sum of complete response (CR) and partial response (PR), and of CR, PR and stable disease (SD), respectively, assessed according to RECIST v1.1 criteria by a NEN-expert radiologist (C.M.). 

### 2.6. Statistical Analysis

Categorical variables were expressed as numbers (percentage), while continuous variables as median and interquartile range [IQR] or mean ± standard deviation (SD), when appropriate. Categorical variables were compared using Pearson’s chi-square or Fisher’s exact test, when appropriate. Continuous variables were compared using Mann–Whitney U test or Student’s *t*-test. Median of PFS and OS were estimated using the Kaplan–Meier method and 95% confidence intervals (95%CI) estimated by the Greenwood formula. Survival outcomes by groups were compared using the restricted mean survival time (RMST) method using the longest follow-up as observation time. The RMST method was applied to overcome the low event rate that is typically observed in studies involving NET patients. ANCOVA-type analyses were used to adjust RMST results for covariates. Cox-proportional hazard regression was used to assess hazard ratios (HR) and 95% confidence interval (95%CI) of factors related to the primary endpoint, namely PFS, and OS. All *p*-values < 0.05 were considered statistically significant. MedCalc Statistical Software version 19 (MedCalc Software, Ostend, Belgium; https://www.medcalc.org) and R version 3.6.1 were used.

## 3. Results

**Figure 1:**
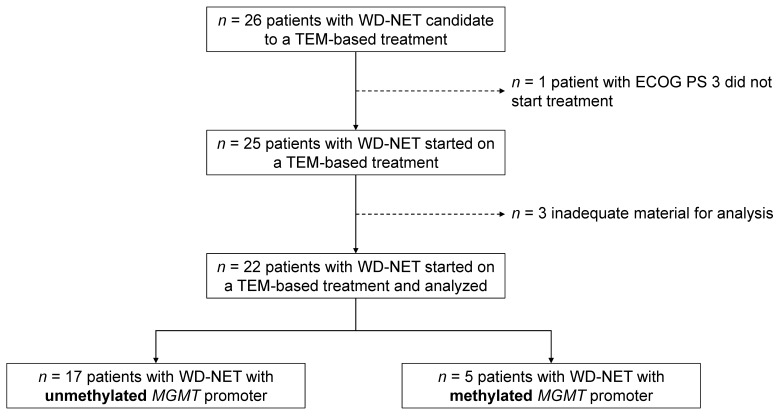
Study flow-chart. WD-NET—well-differentiated neuroendocrine tumors; ECOG PS—Eastern Cooperative Oncology Group performance status; TEM—temozolomide; MGMT—O6-methylguanine-DNA methyltransferase.

Twenty-six patients meeting the inclusion criteria were enrolled in the study. One patient was excluded due to the deterioration of their clinical conditions before the treatment started (screening failure), and three patients were excluded from the analysis due to inadequate material for the MGMT-promoter methylation status assessment (insufficient tissue for DNA extraction or technical problems with the assay). Overall, 22 patients were included in the final analysis (Figure 1).

### 3.1. Baseline Patients’ Characteristics

The baseline patients’ characteristics are summarized in Table 1. Among the final study population (*n* = 22), 13 patients were female (59%) and 9 (41%) male; the median age at enrollment was 64 years (IQR 56–74). Eighteen patients (82%) had an ECOG PS of 0, and four (18%) an ECOG PS of 1. One patient was affected by MEN-1 syndrome and a gastrin-producing panNET. The primary site of the NET was the pancreas in 14 patients (64%), the lung in 5 (23%), and the gastro-intestinal tract in 2 (9%), while in 1 patient, there was a double primary (pancreas and small bowel). Among the five patients with lung NETs, four of them had an atypical carcinoid, and one a typical carcinoid. According to WHO’s 2019 classification, three patients (14%) had a grade-1 tumor, twelve (54%) a grade 2, and seven (32%) a grade 3. The median Ki-67 was 15% (IQR 8–25). The MGMT promoter was methylated in five tumors (23%), all of which were panNETs, accounting for a 33% (5/15) relative frequency of *MGMT*-methylated tumors in this group. The baseline patients’ characteristics by the *MGMT*-methylation status are reported in Table 2.

### 3.2. TEM-Based Treatment

Eleven patients (50%) received CAPTEM and eleven patients (50%) received TEM as monotherapy (Table 3). Of the 11 patients undergoing CAPTEM treatment, 2 discontinued capecitabine but continued the TEM treatment: 1 patient for toxicity and 1 as maintenance treatment after 42 cycles of CAPTEM combination. TEM-based was the first-line treatment in eight cases (36%), the second line in five (23%), and the third or beyond line in nine (41%). The median time to progression observed in the previous treatment line was 7.5 months (95%CI 4–15). The median duration of the TEM-based treatment was 13 months (95%CI 9–34), with a median number of cycles of 12 (IQR 9–22). The median follow-up time was 23 (IQR 13–44) months. At the time of the data analysis (October 15, 2022), 11 patients (50%) were still receiving TEM-based treatment, 2 (9%) discontinued treatment following a long-lasting response, 3 (14%) discontinued treatment for AEs, and 6 (27%) discontinued for PD, of whom 4 had died. In our cohort, three (60%) out of the five MGMT-methylated patients received the doublet therapy (CAPTEM), while two (40%) received temozolomide as a single agent. In the non-methylated group (17 patients), 8 (47%) patients received the doublet therapy, while 9 (53%) received temozolomide alone (Fisher’s *p* = 1.00).

### 3.3. Correlation of MGMT-Promoter Methylation Status with Outcomes

**Figure 2:**
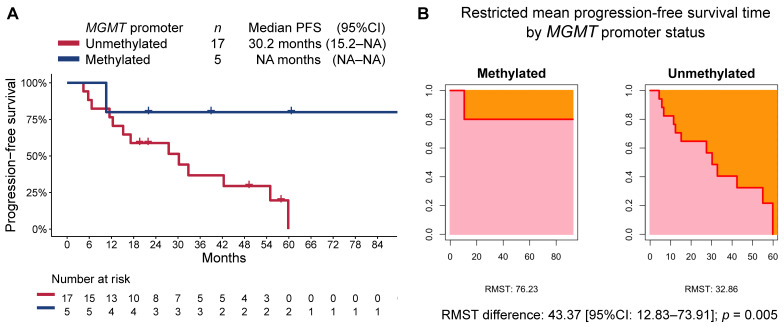
(**A**) Kaplan–Meier estimate of progression-free survival (PFS) by MGMT-promoter methylation status, and (**B**) restricted mean progression-free survival time (RMST) by MGMT-promoter methylation status.

**Figure 3:**
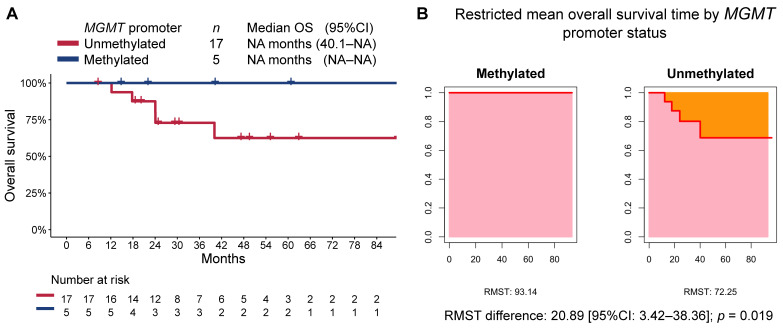
(**A**) Kaplan–Meier estimate of overall survival (OS) by MGMT-promoter methylation status, and (**B**) restricted mean progression-free survival time (RMST) by MGMT-promoter methylation status.

At a median follow-up time of 47.2 months (95%CI 29.3–89.7), the median PFS in the overall cohort was 32.8 months (95%CI 17.2–NA), while the median OS was not reached. Patients in the methylated MGMT group, when compared to those in the unmethylated MGMT group, had longer PFS (median not reached [95%CI NA–NA] vs. 30.2 months [95%CI 15.2–NA], respectively; RMST *p* = 0.005; Figure 2) and OS (median not reached [95%CI NA–NA] vs. not reached [40.1-NA], respectively; RMST *p* = 0.019; Figure 3). The median follow-up time as estimated by the reverse Kaplan–Meier method was 47.2 months (95%CI: 24.8–NA) in the MGMT-unmethylated group and 40.2 months (95%CI: 22.1–NA) in the MGMT-methylated group. 

Since patients with panNETs had longer PFS when compared to those with extra-pancreatic NETs (55.0 months [95%CI 27.5–NA] vs. 30.2 months [6.6–NA], respectively; RMST *p* = 0.016), we sought to correct for potential covariates and found that the MGMT-promoter status retained its association with progression-free survival (adjusted RMST difference: 37.89 [95%CI 3.63–72.16]; *p* = 0.03; Table 4).

In the overall population, the observed best responses were CR in one case (5%), PR in six (27%), and SD in thirteen (59%) for an ORR and DCR of 32% and 91%, respectively. Among the five patients with MGMT-promoter methylation, the best response was CR for one patient, PR for two, and SD for two, while among the seventeen patients in the unmethylated group, the best response was PR in four cases and SD in thirteen. According to the MGMT-promoter status, no significant difference was observed in ORR (60% [95%CI 15–95] vs. 24% [95%CI 7–59]; *p* = 0.274) or DCR (100% [95%CI 48–100] vs. 88% [95%CI 64–99]; *p* = 1.00) in the methylated group compared to the unmethylated MGMT one, respectively.

### 3.4. Exploratory Analysis in Patients with panNET

**Figure 4:**
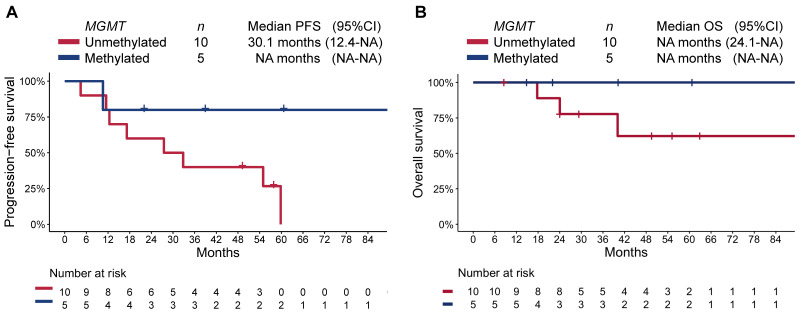
Kaplan–Meier estimate of (**A**) progression-free survival (PFS), and (**B**) overall survival (OS) by MGMT-promoter methylation status in patients with pancreatic NET.

Since pancreas was the most common primary in our cohort (*n* = 15), and given all patients with methylated MGMT promoter belonged to this group, we conducted a subgroup analysis limited to these patients. As in the overall cohort, patients with a methylated-MGMT panNET had longer PFS (not reached [95%CI NA–NA] vs. 32.8 [95%CI 12.4–NA]; RMST *p* = 0.016) and OS (median not reached [95%CI NA–NA] vs. not reached [40.1–NA], respectively; RMST *p* = 0.031; Figure 4).

### 3.5. Safety 

The most commonly reported AEs were fatigue, hematological toxicity (anemia, non-febrile neutropenia, thrombocytopenia), and gastro-intestinal toxicity. Overall, G1-2 AEs were reported in 14 patients (64%), while G3 AEs were reported in 2 cases (9%) (Table 5). No G4 AEs were reported. Three patients (14%) discontinued treatment due to adverse events. Among the three patients discontinuing TEM treatment for AEs, only one had treatment-related toxicities (G3 nausea and diarrhea), while the other two patients stopped treatment because of treatment-unrelated events. One patient discontinued capecitabine due to G3 thrombocytopenia, and continued TEM treatment with no further toxicity. 

### 3.6. Cost Analysis and Feasibility

The costs related to the MGMT-promoter methylation analysis have been evaluated. For each patient, three assays had to be performed: one on the tested tumor sample, one on a non-neoplastic sample, and one on a positive control (a known MGMT-methylated sample). Processing each assay costs around EUR 20, thus the cost for the final analysis of each patient is EUR 60. Overall, for the 22 patients included in this study, the methylation assays have costed EUR 1320, and the number needed to be tested to find a MGMT-unmethylated tumor was 4.4. Since the observed RMST difference for PFS has been 43.37, there has been a 1.97 RMST PFS increase for each assay performed, and an increase of 1 RMST in PFS has been observed every EUR 30.44.

## 4. Discussion

In this study, we prospectively demonstrated that *MGMT*-promoter methylation predicts better survival outcomes to TEM-based chemotherapy in patients with WD-NET, and showed the ability of the *MGMT*-methylation status to predict outcome irrespective of the NET primary site. Furthermore, we have shown that the pyrosequencing assay is feasible and cost-effective. In addition, since the pancreas was the most common primary site and all the *MGMT*-methylated tumors were panNETs, we conducted a subgroup analysis in this group showing that the *MGMT*-methylation status can discriminate patients with better outcome to TEM-based chemotherapy also in patients with panNET.

WD-NETs are mostly slow-growing indolent tumors whose treatment is based on targeting somatostatin receptors with somatostatin analogs or peptide radionuclide receptor therapy [31,32,33,34,35], the mTOR pathway with everolimus [36,37,38,39,40], or neoangiogenesis with sunitinib in panNETs [41], while chemotherapy is usually deferred to later lines. Nevertheless, there are selected cases in which chemotherapy has a prominent role and acquires priority over the aforementioned treatments, such as in large symptomatic tumors or those that need shrinkage to undergo surgery, and in G3 NETs [5,6,7]. In these settings, TEM and CAPTEM are the most-used chemotherapy regimens, given their established activity profile, tolerability, and safety [1,2,3,4,18], but also other fluoropyrimidine-based or oxaliplatin-based regimens are used. Because of its predictive role in gliomas, MGMT deficiency has been advocated as a potential factor that could inform on the best sequencing of treatment in WD-NETs [23]. Nevertheless, conflicting results in patients with NETs, likely related to the different assays used, have somehow limited MGMT-assessment use in the clinic. Indeed, although two retrospective studies did not find a correlation between MGMT deficiency and outcomes to TEM, both assessed MGMT’s protein expression by using IHC [18,19]. Another study assessed both MGMT protein expression and *MGMT*-promoter methylation by using pyrosequencing and found no correlation with the outcomes, but both patients who received TEM and patients who received dacarbazine, another alkylating agent, were included [21], while a series by Kulke et al. found that MGMT IHC was associated with ORR in a cohort of 21 patients with NETs of different origin who received TEM [23]. On the other hand, two studies involving 10 and 43 patients with panNET, respectively, found that the *MGMT*-promoter methylation status was associated with ORR and PFS, respectively [21,22]. 

We previously published one of the largest retrospective, multicenter series on the role of *MGMT*-promoter methylation that included 95 patients with advanced panNET undergoing TEM-based treatment [25]. The *MGMT*-promoter methylation status was assessed by using sequencing techniques (pyrosequencing and methylation-specific polymerase chain reaction), and was independently associated with ORR, PFS, and OS. In this series, the median PFS was 21 and 8 months for *MGMT*-methylated and *MGMT*-unmethylated patients, respectively, while the median OS was not reached and 23 months, respectively. Despite the fact that methylation-specific sequencing techniques, such as the pyrosequencing used in our prospective study, seem to perform better in predicting outcomes to TEM-based chemotherapy as compared to IHC, a recent meta-analysis showed that both MGMT-deficiency assessment methods are associated with ORR to TEM or other alkylating agents in patients with NETs from different primary sites [24]. 

Recently, data from the ECOG-ACRIN E2211 study, the first prospective, multicenter, randomized phase II trial comparing TEM (*n* = 65) and CAPTEM (*n* = 68) in patients with panNET, have been reported [42]. PFS, the primary endpoint, was longer in the CAPTEM arm than in the TEM arm (22.7 vs. 14.4 months, respectively; *p* = 0.022; HR: 0.58), while OS (58.7 vs. 53.8 months, respectively; *p* = 0.42) and ORR (39.7% vs. 33.8%; *p* = 0.59) were not significantly different between the two arms. However, tumor grade, which is a known prognostic factor in NETs, was not a stratification factor at randomization, and there was a predominance of grade-2 tumors in the TEM arm. Interestingly, this study showed that both MGMT IHC and *MGMT*-promoter methylation were associated with ORR, irrespective of treatment. A French prospective, multicenter, open-label, randomized phase II trial (MGMT-NET; NCT03217097) randomized patients with NET to receive TEM-based or oxaliplatin-based treatments stratified by *MGMT* methylation. The study, whose primary endpoint is ORR at 3 months based on *MGMT* methylation on tumor tissue, has completed enrolment and results are being awaited [26]. 

In our cohort, the doublet regimen (CAPTEM) was received by 60% of MGMT-methylated patients and 47% of the unmethylated ones. Despite there not being any formal difference between the two groups, the small sample size does not allow to definitively rule out a possible, although unlikely, influence of the two different regimens (doublet vs. monotherapy) in the two groups.

We also acknowledge that there is considerably lower evidence on the efficacy of CAPTEM association in lung and small bowel NETs, while in pancreatic NETs, the doublet regimen has shown a greater benefit compared to temozolomide in monotherapy [17]. In our cohort, to limit a possible influence of the primary site on the results, we have conducted a sub-group analysis restricted to panNETs. In the panNET subgroup, despite the small number of patients (15, of which 5 presented MGMT methylation), we observed that PFS and OS were significantly longer in the methylated group compared to the unmethylated group. In keeping with this, the primary site (pancreatic vs. other) did not affect PFS in the adjusted RMST difference analysis. In a recent paper by Della Monica et al. [42], on 42 NENs of different primary sites and grading, the authors reported that MGMT was substantially more often methylated in G1 and G2 NETs (76% of cases) compared to G3 NENs (62% of cases), although the data were not statistically significant probably due to the low number of cases. In particular, the authors reported a trend towards a higher degree of methylation for the differentiated tumor group when analyzing each CpG site covered by methylation-specific PCR. Differently from what was reported by Della Monica et al., in our cohort, MGMT-methylated patients had G3 tumors more often, although they were well differentiated. In detail, 13 out of 17 (76%) unmethylated patients and 2 out of 5 (40%) methylated patients had a G1-G2 NET. However, no direct comparison with the above-mentioned cohort should be made due to the different methods used for MGMT methylation analysis in the two cohorts and to the small number of patients.

In this study, we acknowledge some limitations, including the relatively small samples size. Nevertheless, a substantial part of the study has been conducted during the COVID-19 pandemic, which negatively affected the accrual rate, as many patients may have not been referred from other centers in this period due to the international emergency state and the travel limitations. In our overall population, ORR, PFS, and OS compared favorably to the outcomes reported in other studies evaluating TEM-based treatment activity [2,25,43], which may suggest that our cohort is comparable to the general population of patients with NET. Additionally, all tumors in the methylated *MGMT*-promoter group were panNETs. Nevertheless, panNETs are the most frequent advanced WD-NET [44], have a more aggressive course compared to those of lung or small-bowel primary, and have the most solid evidence of response to TEM-based treatments. These considerations explain why the majority of patients with WD-NET who were candidates by clinical practice to receive TEM-based chemotherapy had a panNET. Furthermore, the higher prevalence of *MGMT*-promoter methylation reported in panNETs when compared to NETs of other sites accounts for the fact that all tumors in the methylated *MGMT*-promoter group had a panNET [22,23,45,46,47]. As a consequence, the findings of our study are more likely applicable only to panNETs treated with TEM, rather than to NETs from other sites.

The loss of *MGMT* function can also be caused by mechanisms other than promoter methylation, such as gene copy loss. However, our aim was to investigate the role of a rapid, reproducible, inexpensive, and widely available test, such as methylation-specific promoter pyrosequencing, in order to reason about potentially implementing it in clinical practice, which, however, does not allow us to assess gene copy-number variations.

Previous studies have investigated the performance of other methods to assess MGMT status, especially in the glioblastoma setting. In 2016, Cives et al. reported that MGMT status by IHC failed to predict the response to CAPTEM in 143 pancreatic NET patients; MGMT status by IHC was not prognostic either [19]. It is also known that IHC is often burdened by several pitfalls such as sample bias, sampling issues, interobserver variability, and technical differences (including the use of different antibodies against MGMT), while the interpretation of pyrosequencing can be considered less prone to operator-dependent subjectivity and related limitations [48]. Several other studies have reported that IHC is associated with a poor reproducibility and a high interobserver variability. Furthermore, individual MGMT proteins can be reduced upon interaction with the appropriate substrate and, thus, the amount of protein detected may not be a reliable expression marker. In conclusion, the evidence available to date, although scarce and based on glioblastoma patients, is that, despite its simplicity, MGMT determination by IHC is not sufficiently reliable for basing management decisions upon [49,50].

Lastly, in our previous retrospective study on 95 patients with advanced NENs undergoing treatment with TEM-based therapy, we reported that the MGMT-promoter methylation status evaluated by using pyrosequencing could effectively predict treatment response [25].

In keeping with these observations, when we designed the prospective trial, we decided to investigate the role of the most convenient, easily reproducible, and widely available test, such as pyrosequencing, and, ultimately, to prospectively validate the observations of our previous retrospective study.

## 5. Conclusions

This study has prospectively demonstrated the role of the MGMT-promoter methylation status as a predictive factor for TEM-based treatment response in patients with advanced WD-NETs, as well as its feasibility and cost-effectiveness. Our findings support the use of the MGMT-promoter methylation status to guide treatment selection in this setting. Due to our patient population composition, these results should be applied in patients with panNET and, with caution, in patients with NET from other primary sites.

## Figures and Tables

**Table 1:** Patients’ characteristics.

Characteristic		*n* (%)
Overall		22 (100%)
Gender (female)		13 (59%)
Age (years), median (IQR)		64 (56–74)
ECOG PS 0		18 (82%)
MEN-1 syndrome		1 (5%)
Functioning NET		1 (5%)
Primary tumor site		
Pancreas:		14 (64%)
	Head	7
	Body	4
	Tail	3
Lung:		5 (23%)
	Atypical carcinoid	4
	Typical carcinoid	1
Gastro-intestinal:		2 (9%)
	Stomach	1
	Ileum	1
Double site (pancreas and ileum)		1 (5%)
WHO’s 2019 grading:		
	G1	3 (14%)
	G2	12 (54%)
	G3	7 (32%)
Ki-67 (%), median (IQR)		15 [8,9,10,11,12,13,14,15,16,17,18,19,20,21,22,23,24,25]
*MGMT*-promoter methylation		5 (23%)

IQR—interquartile range; ECOG PS—Eastern Cooperative Oncology Group performance status; WHO—World Health Organization; G—grade; MGMT—O6-methyl-guanine-DNA methyl-transferase.

**Table 2:** Patients’ baseline characteristics by *MGMT*-promoter methylation status.

Characteristic		Unmethylated MGMT	Methylated MGMT
Overall		17	5
Gender (female)		11	2
Age (years), median (IQR)		66 (56–73)	74 (60–77)
ECOG PS 0		14	4
MEN-1 syndrome		1	0
Functioning NET		1	0
Primary tumor site			
Pancreas:		9	5
	Head	4	3
	Body	3	1
	Tail	2	1
Lung:		5	0
	Atypical carcinoid	4	
	Typical carcinoid	1	
Gastro-intestinal:		2	0
	Stomach	1	
	Ileum	1	
Double site (pancreas and ileum)		1	0
WHO’s 2019 grading:			
	G1	3	0
	G2	10	2
	G3	4	3
Ki-67 (%), median (IQR)		15 (7–22)	25 (6–41)
Treatment regimen:			
	TEM	9	2
	CAPTEM	8	3

IQR—interquartile range; ECOG PS—Eastern Cooperative Oncology Group performance status; WHO—World Health Organization; G—grade; MGMT—O6-methyl-guanine-DNA methyl-transferase; TEM—temozolomide; CAPTEM—capecitabine and temozolomide doublet.

**Table 3:** Treatment characteristics.

Characteristic	*n* (%)
Overall	22 (100%)
Treatment regimen	
TEM	11 (50%)
CAPTEM	11 (50%)
Duration (months), median (95%CI)	46.1 (12–NA)
Number of cycles, median (IQR)	12 (9–22)
Ongoing treatment	11 (50%)
Treatment line	
First	8 (36%)
Second	5 (23%)
Third or beyond	9 (41%)
TTP to previous treatment line (months), median (95%CI)	7.5 (5–20)

TEM—temozolomide; CAPTEM—capecitabine and temozolomide; IQR—interquartile range; TTP—time to tumor progression.

**Table 4:** Unadjusted and adjusted RMST difference for progression-free survival.

Variable	Unadjusted			Adjusted		
	RMST	95%CI	*p*	RMST	95%CI	*p*
Sex (M vs. F)	9.28	−10.27–28.83	0.352			
Age (≤ vs. > median)	4.44	−13.25–22.13	0.623			
ECOG PS (1 vs. 0)	0.69	−4.86–6.24	0.809			
Primary (pancreas vs. other)	26.38	4.84–47.91	**0.016**	16.19	−3.06–35.44	0.099
Grading (G3 vs. G1-2)	23.43	−7.90–54.75	0.143			
Ki67 (> vs. ≤ median)	21.43	−5.11–47.96	0.113			
Regiment (CAPTEM vs. TEM)	3.79	−14.80–22.38	0.689			
Treatment line (1st vs. ≥ 2nd)	2.19	−17.35–21.73	0.826			
*MGMT*-promoter methylation (methylated vs. unmethylated)	43.37	12.83–73.91	**0.005**	37.89	3.63–72.16	**0.030**

Bold highlight significant p-values.

**Table 5:** Treatment-related adverse events.

Adverse Event	Any Grade	G1-2 (%)	≥G3 (%)
Any adverse event	16 (73%)	14 (64%)	2 (9%)
Fatigue	4 (18%)	4 (18%)	0
Thrombocytopenia	4 (18%)	3 (14%)	1 (4%)
Neutropenia	1 (4%)	1 (4%)	0
Anemia	2 (10%)	2 (10%)	0
Gastro-intestinal	5 (23%)	4 (18%)	1 (4%)

G—grade according to common toxicity criteria for adverse events (CTCAE) v.5.0.

## Data Availability

The data presented in this study are available on request from the corresponding author.
